# Earlier Return to Soccer After Anterior Cruciate Ligament Reconstruction and Concomitant Meniscal Repair Predict Better Outcomes 3 Years Postoperatively

**DOI:** 10.1007/s43465-025-01588-y

**Published:** 2025-12-22

**Authors:** Filippo Familiari, Filippo Anghilieri, Michele Mercurio, Giorgio Gasparini, Robert F. LaPrade, Corrado Bait

**Affiliations:** 1 Department of Orthopaedic and Trauma Surgery, Magna Graecia University, V.Le Europa (Loc. Germaneto), 88100 Catanzaro, Italy; 2 Research Center On Musculoskeletal Health, MusculoSkeletalHealth@UMG, Magna Graecia University, Catanzaro, Italy; 3 Division of Orthopaedic and Trauma Surgery, Villa Aprica Clinical Institute, Como, Italy; 4 Twin Cities Orthopedics, Minneapolis, MN USA

**Keywords:** ACL reconstruction, Hamstring, Soccer, IKDC, ACL-RSI

## Abstract

**Objectives:**

The aim of this study was to investigate functional and psychological outcomes to identify successful return to sport (RTS) predictors after arthroscopic meniscal repair with concomitant anatomic single-bundle ACL reconstruction (ACLR) in soccer players.

**Methods:**

Forty-three out of forty-six soccer players who underwent primary unilateral arthroscopic anatomic single-bundle ACLR with concomitant meniscal repair were available at follow-up. Postoperatively, knee function, generic health outcomes, and psychological impact were assessed using the IKDC, the SF-12, and the ACL return to sports after injury (ACL-RSI) scale.

**Results:**

After a mean follow-up of 34 ± 15.1 months, 82% of soccer players returned to their previous level of play. The mean time to RTS was 7.8 ± 1.6 months (range 6–12 months). A positive strong correlation between IKDC versus ACL-RSI (*p* < 0.001) was noted. The correlation was moderately positive between IKDC versus SF-12 PCS (*p* < 0.001), ACL-RSI versus SF-12 PCS (*p* < 0.001), ACL-RSI versus SF-12 MCS (*p* < 0.001), and weakly positive between IKDC versus SF-12-MSC scores (*p* < 0.001). Higher postoperative ACL-RSI scores were directly related to younger age (*p* = 0.036) and shorter RTS time after surgery (*p* = 0.027). The multivariate analysis confirmed the association between a higher postoperative ACL-RSI and a shorter RTS time after surgery (*p* = 0.043). Higher postoperative SF-12 PCS values were affected by a shorter RTS time after surgery (*p* = 0.005).

**Conclusion:**

Higher postoperative ACL-RSI scores were directly related to younger patient age and shorter RTS time after surgery.

**Level of evidence:**

III.

## “What are the New Findings”

- Soccer players returning earlier to the sport show better physical and psychological results.
- Higher postoperative ACL-RSI scores are directly related to younger patient age and shorter RTS time after surgery.
- Higher postoperative SF-12 PCS values are affected by a shorter RTS time after surgery.

## Introduction

Pivoting sports such as soccer are considered high-risk activities for anterior cruciate ligament (ACL) non-contact and contact injuries [1, 2] because of the high axial and torsional loads applied to the knee joint during sport-specific tasks [3], such as sudden change of direction, rapid deceleration, landing from a jump and contact with an opponent. Soccer is the most played sport in the world, with an estimated 265 million active soccer players [4], and the Union of European Football Associations (UEFA) has expressed its concern over the physical and mental load on professional soccer players and the associated possible risk of injury. The ACL injury rate is 0.4–0.6 per soccer team every season, and more than 120,000 individuals with ACL injury undergo reconstruction annually in the United States [5]. The burden of ACL injury is particularly relevant since players lose at least 6 months of play on average, and the long-term consequences, including early onset osteoarthritis, are well known [6]. Currently, ACL reconstruction (ACLR) is highly recommended in patients who must perform at a high physical level [7]. Indeed, one important goal for ACLR is to quickly return to sport (RTS) at the same intensity level as that before the injury occurred [8]. Even if most players can return to soccer after ACLR, time-loss injuries that result in long periods without training or competing are considered major adverse events for the career of a soccer player, which in the worst case may lead to early retirement [9, 10]. Moreover, Kilic et al. [11] found that athletes who suffered from severe musculoskeletal time-loss injuries are likely to develop subsequent symptoms of mental disorder, such as distress, anxiety, and depression, emphasizing the need for an interdisciplinary medical approach. Therefore, a combination of functional and psychological outcome measures is likely necessary to provide a comprehensive evaluation of RTS after ACLR [12]. Previous studies [13, 14] have found that the key factors that increase the possibility of a successful return to a preinjury level of sport after ACLR include younger age, male gender, playing an elite sport, and a positive psychological response.

The purpose of this study was to investigate functional and psychological outcomes to identify successful RTS predictors after arthroscopic meniscal repair with concomitant anatomic single-bundle ACLR. It was hypothesized that soccer players treated with ACLR and concomitant meniscal repair will have a high percentage of return to sport. Further, those who return to sport sooner within a pre-determined safe window after surgery will experience better psychological and subjective outcome scores.

## Materials and Methods

The study protocol was approved by the local Ethics Committee (Institutional Review Board approval was obtained from Magna Graecia University of Catanzaro, ID number 87/18), and the research was conducted in compliance with the Declaration of Helsinki and in accordance with the STROBE guidelines.

A retrospective multicenter study with prospective data collection was undertaken on 54 patients who underwent primary unilateral arthroscopic meniscal repair with a concomitant anatomic single-bundle ACLR between October 2016 and September 2021 at the following institutions: Mater Domini University Hospital of Catanzaro (Italy) and Villa Aprica Clinical Institute of Como (Italy).

### Patient Involvement Statement

All participants had a minimum of 6 months postsurgical recovery at the time of participating in the study. The 6-month time point corresponds to RTS defined as the return to full training and matches with the team without restrictions. Eligibility criteria were as follows: (1) ≥ 16 years old at the time of surgery, (2) all-inside repair for meniscal tear, (3) ACLR using semitendinosus and gracilis tendon autograft (ST-G), (4) played soccer at least once a week in training or in a match prior to the injury, (5) return to full participation in soccer after surgery, and (6) the capability to communicate with health care professionals and give valid informed consent. Soccer players who underwent bilateral meniscal repair or ACLR, revision surgery, multi-ligament injuries, and fractures of the knee at any time as well as patients showing severe knee osteoarthritis (Kellgren–Lawrence grade III and IV) were excluded. The diagnosis was based on clinical and radiological evaluation of the knee (plain radiographs and magnetic resonance imaging). A total of 46 patients met the study criteria. Signed informed consent was provided by each patient who was made aware of all aspects of the study [2]. Demographic data, including age, gender, mechanism of injury (contact or non-contact), dominant limb defined as the preferred kicking limb, level of play (professional or nonprofessional), and playing surface (natural grass and artificial turf), were assessed.

### Surgical Technique

All surgical procedures were performed by two surgeons (FF and CB) with high levels of experience in knee arthroscopy. Meniscal tears were repaired using the all-inside technique. For the tibial tunnel for the ACLR, the aimer was adjusted to the 45°–55° position to ensure adequate tibial tunnel length, and the guide tip was positioned intraarticularly through the anteromedial portal. Femoral tunnel drilling was performed via the anteromedial portal technique with the knee flexed to 120°; tunnel positions were placed within the native ACL footprints. The ST-G were harvested for a 4-strand graft aiming for a minimum graft diameter of 8 mm. In all ACLR procedures, a cortical suspension device was used for femoral fixation. A bioabsorbable interference screw was used for tibial fixation while the graft was tensioned at 30° of knee flexion.

### Postoperative Rehabilitation

Postoperatively, patients walked with elbow crutches bearing weight as tolerated, and the range of motion was limited to 90° for 1 week. After 1 week, patients began range of motion therapy. After nearly full range of motion was achieved, patients started strength training, with an emphasis on closed kinetic chain exercises. The preservation of quadriceps function in the early postoperative stage of rehabilitation after surgery was emphasized with an early initiation of isometric quadriceps setting exercises. Activities that prepare the individual for progression to full weight-bearing and ambulation, improving balance and postural control, and achieving normal gait were allowed after 4 weeks. Weight-bearing strengthening activities were initiated and progressed after 6 weeks. Strengthening exercises through the full range of motion and activities to enhance neuromuscular control were also advanced to ensure full recovery and maintenance of strength and dynamic stability. Rehabilitation progressed from functional activities to sports-specific training. When the quadriceps limb symmetry index was 85% or greater and strength, proprioception, and endurance for functional progression were satisfied, the patients began full-effort sprinting, cutting, and plyometric activities. RTS was permitted no earlier than 6 months after surgery.

### Patient-Reported Outcome Measures

At follow-up, each patient was functionally and psychologically evaluated by a trained researcher. Measures of knee function and generic health outcomes were assessed using the International Knee Documentation Committee subjective knee form (IKDC) and the 12-Item Short Form Survey (SF-12), respectively.

The IKDC [15] is an 18-item instrument designed to measure pain, symptoms, function, and sports activity. Possible score range from 0–100, where 100 means no limitation with daily or sporting activities and the absence of symptoms.

The 12-Item Short Form Survey (SF-12) [16] is a 12-item questionnaire used to assess generic health outcomes from the patient’s perspective. Two summary scales (the physical and the mental component summary, PCS and MCS, respectively) can be computed with scores ranging from 0 to 100%, with higher scores indicating a better quality of life.

The psychological impact was assessed using the ACL return to sports after injury (ACL-RSI) scale developed and validated by Webster et al. [17, 18] in relation to three elements that have been correlated with RTS in the literature: emotions, confidence in one’s performance, and the evaluation of risk. The total score was obtained by adding the values of the 12 responses and then obtaining their relationship to 100 to obtain a percentage. High scores corresponded to a positive psychological response.

### Statistical Analysis

The mean, standard deviation, and range were noted for the continuous variables, and counts were noted for the categorical variables. All data were collected, measured, and reported with one decimal accuracy. The distribution of the numeric samples was assessed with the Kolmogorov–Smirnov normality test. Based on this preliminary analysis, parametric tests were adopted. Correlations between outcome scales with pairwise correlation coefficients were evaluated; the correlation was considered to be strong (*r* > 0.5), medium (0.5 < *r* < 0.3), or small (0.3 < *r* < 0.1) [19]. The RTS rate was calculated as the percentage of surgical treated players who returned to soccer training and match to their previous level of play [20]. Uni- and multivariate linear regression were performed on the whole population to test possible outcome predictors. The explanatory and confounding pre- and postoperative variables included in the analysis were age (continuous), sex (categorical), follow-up (continuous), body mass index (BMI) (continuous), dominant limb (categorical), playing surface (categorical), level of sport (categorical), meniscus repaired (categorical), and mechanism of injury (categorical). Only explanatory and confounding variables that showed a trend toward an association (e.g., *p* < 0.10) with the outcome of interest in the univariate analysis were inserted in the multiple regression analysis.

Post hoc power was calculated by considering IKDC as main point with a minimal clinically important difference of 13.8, the sample size, the observed effect size, and an *α*-value of 0.05; a post hoc power greater than 80% was considered appropriate. IBM SPSS Statistics software (version 26, IBM Corp., Armonk, NY, USA) and GraphPad Prism (version 7.0, GraphPad Software Inc., San Diego, CA, USA) were used for the database construction and statistical analysis. Confidence intervals (Cis) were set at 95%, and a *p* value less than 0.05 was considered significant.

## Results

Of the initial 46 patients, 3 were lost at follow-up and excluded from further analysis, leaving 43 patients (93.5%) available for the final evaluation. The characteristics of the study population are summarized in Table 1.

**Table 1:** Baseline characteristics of included patients

Patients (no. = 43)	Mean ± SD (range) or no. (%)
Gender	
Male	28 (65.1%)
Female	15 (34.9%)
Age at surgery (years)	28 ± 10.6 (16–50)
Dominant limb	24 (55.8%)
Level of play	
Professional	11 (25.6%)
Non-professional	32 (74.4%)
BMI (kg/m^2^)	22.9 ± 3.1 (18–30)
Playing surface	
Natural grass	13 (30.2%)
Artificial turf	30 (69.8%)
Mechanism of ACL injury	
Contact	12 (27.9%)
Non-contact	31 (72.1%)
Meniscus sutured	
Medial	16 (37.2%)
Lateral	24 (55.8%)
Medial and lateral	3 (7%)
Follow-up (months)	34 ± 15.1 (10–65)

BMI, means body mass index; ACL, anterior cruciate ligament; SD, standard deviation; No., number of cases

There were 28 (65.1%) men, and the mean age at surgery was 28 ± 10.6 years (range, 16–50 years). Professional soccer players were 11 (25.6% of the cohort), and the dominant limb was involved in 55.8% of cases. The mean time to RTS was 7.8 ± 1.6 months (range, 6–12 months). Overall, 82% of patients returned to their previous level of play.

Clinical outcomes after a mean follow-up period of 34 ± 15.1 months are reported in Table 2.

**Table 2:** Clinical outcomes of study population

	Mean	Standard deviation	Min	Max
SF-12-PCS	51.5	4.1	35.4	57.4
SF-12-MCS	52.2	7.7	27.1	59.7
IKDC	74.5	12.0	18.4	94.3
ACL-RSI	74.6	14.8	15.0	93.0

SF-12 means, 12-Item Short Form Survey; PCS, physical component score; MCS, mental component score; IKDC, International Knee Documentation Committee subjective knee form; ACL-RSI, anterior cruciate ligament return to sports after injury

Table 3 demonstrates the correlation analysis among outcome measures. The correlation was positively strong between IKDC versus ACL-RSI (*r* = 0.701; *p* < 0.001). The correlation was moderately positive between IKDC versus SF-12 PCS (*r* = 0.419; *p* < 0.001), ACL-RSI versus SF-12 PCS (*r* = 0.387; *p* < 0.001), ACL-RSI versus SF-12 MCS (*r* = 0.363; *p* < 0.001), and weakly positive between IKDC versus SF-12-MSC scores (*r* = 0.290; *p* < 0.001).

**Table 3:** Correlation analysis among outcome measures

	SF-12	SF-12	IKDC
	PCS	MCS	
SF-12 MCS	0.024 (*p* = 0.160)		
IKDC	**0.419 (*p* < 0.001)**	**0.290 (*p* < 0.001)**	
ACL-RSI	**0.387 (*p* < 0.001)**	**0.363 (*p* < 0.001)**	**0.701 (*p* < 0.001)**

Highlighted in bold results that had a p value <0.05 and therefore a statistical significance

SF-12 means, 12-Item Short Form Survey; PCS, physical component score; MCS, mental component score; IKDC, International Knee Documentation Committee subjective knee form; ACL-RSI, anterior cruciate ligament return to sports after injury

As shown in Table 4, higher postoperative ACL-RSI scores were directly related to younger patient age (*p* = 0.036, *β* = − 0.08) and shorter RTS time after surgery (*p* = 0.027, *β* = − 0.09). The multivariate analysis confirmed the association between a higher postoperative ACL-RSI and a shorter RTS time after surgery (*p* = 0.043, *β* = − 2.172). Higher postoperative SF-12 PCS values were affected by a shorter RTS time after surgery (*p* = 0.005, *β* = − 0.158).

**Table 4:** Univariate analysis

	SF-12-PCS		SF-12-MCS		IKDC		ACL-RSI	
	*Β*	*p* value	*β*	*p* value	*β*	*p* value	*β*	*p* value
Age		0.074		0.118		0.095	** − 0.08**	**0.036**
Sex		0.300		0.499		0.906		0.857
Follow-up		0.166		0.995		0.271		0.057
Return to sport (months)	** − 0.158**	**0.005**		0.403		0.190	** − 0.09**	**0.027**
BMI		0.987		0.637		0.726		0.592
Level of play								
Professional		0.391		0.444		0.703		0.267
Non-professional								
Mechanism of injury								
Contact		0.563		0.948		0.601		0.941
Non-contact								
Playing surface								
Natural grass		0.182		0.102		0.893		0.512
Artificial turf								
Meniscus sutured								
Medial		0.738		0.875		0.296		0.295
Lateral								
Medial and lateral								
Dominant limb involved								
Yes		0.529		0.396		0.851		0.831
No								

SF-12 means 12-Item Short Form Survey; PCS physical component score; MCS mental component score; IKDC, International Knee Documentation Committee subjective knee form; ACL-RSI, anterior cruciate ligament return to sports after injury; SD, Standard Deviation; BMI, body mass index

## Discussion

The most important finding of the present study was that 82% of soccer players undergoing arthroscopic meniscal repair with concomitant ACLR returned to sport at their preinjury level. The mean time to RTS was 7.8 ± 1.6 months (range 6–12 months). In addition, soccer players returning earlier to sport demonstrated better physical and psychological results, and younger patient age. In addition, a shorter RTS correlated with higher postoperative ACL-RSI scores.

The observed RTS rate of 82% was consistent with previously published data, such as a systematic review by Lai et al. which reported a RTS rate of 85% (*n* = 220) for elite-level soccer players [21], and a retrospective review by Forsythe et al. which reported a return rate of 80% (*n* = 41) for UEFA professional soccer players [22]. The mean RTS time for the current study was also comparable to previous results, as Lai et al. found an average RTS time for soccer players between 6 and 10.2 months postoperatively [21].

Of note, Forsythe et al. reported RTS rates after ACLR to be shorter in soccer than in other major sports leagues, although the re-rupture rates were comparable to other sports, suggesting the RTS timing remains appropriate for these athletes [22]. Several factors may have influenced the return to sport rate in our sample, such as level of performance, dominant limb injury, and patient age. In the current study, 11 of the 43 patients are professional-level athletes. While there may be many reasons for this, such as greater rehabilitative support or higher pressure to return, an increased level of play has previously been shown to shorten RTS time. Szymski et al. assessed professional, semi-professional, and amateur soccer players, finding a significant negative correlation between level of play and RTS rate/time [23]. Age continues to be an important part of the RTS conversation as well. Brophy et al. stated that younger soccer players are more likely to return to play after ACLR [24]. In addition, Lentz et al. reported a similar trend across all athletes following ACLR, where the mean age of those returning to sport versus those not returning to sport was 20.9 and 24.2, respectively [25]. While a younger age may increase the RTS rate, it has also been correlated with a greater rate of re-injury, which requires consideration when deeming patients ready to RTS [26–28]. There has been a more recent push to increase the recovery timeline for athletes recovering from an ACLR, given data suggesting increased failure rates when patients return at less than 9 months [29]. Furthermore, there are MRI and clinical data suggesting that the ACL graft does not mature until closer to 12 + months following surgery [30, 31]. However, there is also data which demonstrates that an earlier return to sport is associated with improved physical and psychological results. In the present cohort, a shorter RTS time was significantly associated with higher ACL-RSI and SF-12 PCS scores, demonstrating the efficacy of these assessments in measuring rehabilitation outcomes [32]. This suggests that in some instances, the risk of re-rupture may need to be weighed against the psychological and potentially physical implications on delaying RTS. As well as in early RTS, younger patient age was also found to be correlated, although weakly, with higher postoperative ACL-RSI scores. This finding is consistent with previous literature as well [17, 33].

Regarding patient-reported outcomes, the IKDC specifically has been found in prior literature to be significantly correlated with increased RTS [25], and specific to soccer, several studies have assessed IKDC scores in postoperative ACLR patients [34–36]. While Fälström et al. reported a mean IKDC of 80.2 at final follow-up [34], other studies have higher values, such as Allen et al. and Angelozzi et al. with mean IKDC scores of 95 and 93, respectively [35, 36]. The mean IKDC score of 74.5 in the cohort presented in our study is notably lower than the previous literature described above, which may affect the overall RTS rate of this study. Contrastingly, the mean ACL-RSI score of 74.6 was in line with or greater than previous literature [18, 32, 37]. Manara et al., for example, reported a mean ACL-RSI of 68 for soccer players who returned to sport after ACLR [32], and Langford et al. reported that a ACL-RSI value of 60 or greater was deemed appropriate psychological readiness to return to sport activity [37]. The mean ACL-RSI score, therefore, indicates patients, on average, experienced successful psychological rehabilitation in preparing to resume sport activity. This higher value may help compensate for a lower IKDC score when assessing overall RTS rates against other studies.

Further, the data found a strong positive correlation between IKDC and ACL-RSI scores in this cohort which indicates that subjective knee function and psychological readiness to return to sport were closely connected. This outcome was in line with several previous studies across sports [38–41]. In addition, it has previously been found that athletes who do not RTS at preinjury level have significantly lower ACL-RSI and IKDC scores at end of follow-up [38]. This finding highlights the importance of proper postoperative rehabilitation as a course for not only improving knee function but also psychological readiness.

Our study was not without limitations. While this is a multicenter study with prospective data collection, the small sample size may enhance outliers when assessing mean IKDC and ACL-RSI values. IKDC values ranging widely from 18.4 to 94.3 may explain the low mean IKDC score when compared to the previous literature. A larger sample size may provide a more representative mean IKDC score and higher quality results when assessing IKDC in soccer players returning to sport. In addition, while there was no statistical significance between meniscus sutured (medial vs. lateral) and the SF-12, IKDC, or ACL-RSI assessments, further evaluation is needed to understand meniscal injury in concomitant ACLR with its effect on RTS in soccer players. Beneito Pastor et al. previously looked at return to sport after ACLR and did not find significant differences between mean ACL-RSI scores and the presence or absence of meniscal injury (67.9 vs. 78.2; *p* = 0.13) [38]; however, various types of meniscus tears may change RTS outcomes and ACL-RSI scores which should be assessed further. This is similar to the mechanism of injury, where subcategories of contact and non-contact injuries may delineate statistical differences in mean SF-12, IKDC, or ACL-RSI values.

The strengths of this study expand beyond its prospective data collection. All surgeries were performed by two surgeons (FF and CB) which minimizes potential variations in surgical technique, and utilization of a standardized rehabilitation protocol across all participants helped control for confounding factors and provide uniformity. Moreover, the study included a commendable follow-up duration, on average being 34 months, with sufficient time for long-term evaluation and assessment of physical and psychological rehabilitation. All patients also underwent ACLR with semitendinosus and gracilis tendon autografts, minimizing variation in graft type. While this limits confounding variables, it is worth noting that other autograft choices should be evaluated for their effect on SF-12, IDKC, ACL-RSI, and RTS rates in soccer players. Of note, DeFazio et al. reported on a systematic review and meta-analysis a higher RTS rate when using bone–patellar tendon–bone autograft over hamstring tendon autograft [42].

## Conclusion

After arthroscopic meniscal repair with concomitant anatomic single-bundle ACLR, 82% of soccer players can expect to return to playing sports at their preinjury level. The findings of this study suggest there is a relationship between earlier return to sport and improved physical and psychological outcome score. Higher postoperative ACL-RSI scores were directly related to younger patient age and shorter RTS time after surgery.

## Data Availability

All data underlying the results are available as part of the article and no additional source data are required.
